# A Dream of Health

**Published:** 1921-09-17

**Authors:** 


### A DREAM OF HEALTH.

Shortly before the House of Commons adjourned the Welsh Consultative Council to the Ministry of Health, under the chairmanship of a layman, Sir Edgar Jones, M.P., laid upon the table their report as to the administrative machinery to be desiderated to work their health and medical scheme for Wales and Monmouth. We say desiderated, because no expectation exists of the possibility of bringing such machinery into existence in the near future; in fact, some hesitation was felt in incurring the expense of printing the report. In such circumstances, with the scheme worth little more in practice than the paper on which it is set out, lengthy comment is obviously unnecessary. The essential feature is to do away with the Poor-Law system, dividing its duties between the Ministry of Labour (casuals and tramps) and the Ministry of Health; a change long foreshadowed, by which the care of the sick, the aged, and children, pauper or illegitimate, would come under more favourable auspices.

There is postulated a national health authority or council subject to the Minister of Health; this council to appoint local and regional sub-committees. Its *personnel* is to be constituted as to two-thirds by nominees of county boroughs, county councils, urban and rural district councils with populations exceeding 40,000; and as to one-third by nominees of the Minister of Health. The sub-councils can appoint executive committees (to which all functions save those of finance may be delegated) and also advisory bodies consisting of persons with special experience. Provisos are made for female representation upon all these associations. In a minority report reservations are made as to direct popular election instead of election from the sanitary authorities.

Generally, the report finds a great need for increased accommodation for residential treatment of sick persons. It is pointed out that some of this demand could be met by utilising unused smallpox hospitals. Fever hospital accommodation should be increased to at least one bed for every 1,000 of the population in the populous areas, and one for every 4,000 in the thinly settled districts. Rest or holiday homes are mentioned as advisable. Reference is made to Wales' tuberculosis scheme under the National Memorial Association. There is, however, an unfortunate precedent here; for the most responsible professional position has been given to a military medical man who cannot have had adequate experience of the disease. A complete network of medical institutions for Wales is suggested, the centre of the web to be at Cardiff, in connection with the Welsh National Medical School. The weak point of the scheme is the placing of financial responsibility upon existing local authorities, who are therefore, very naturally, to be given a majority in representation. Who pays the piper must call the tune, of course; but in any health scheme control should be centralised and not placed at many peripheral points.

